# Supplementary material for: Polyketide synthase gene clusters in the ericoid mycorrhizal fungus Oidiodendron maius: genome-wide prediction, expression profiling, and functional characterization of knockout mutants
Source: Front Plant Sci. 2026 Sep 16;17:1924430. doi: 10.3389/fpls.2026.1924430 (PMC13623903; doi:10.3389/fpls.2026.1924430)
Supplement: Supplementary file 1 [file SupplementaryFile1.zip › Supplementary Materials/Supplementary Folder 1.zip!/Oidma1_AssemblyScaffolds_Repeatmasked/knownclusterblast/cluster49/jgi.p_Oidma1_184531_mibig_hits.html]

| MIBiG Protein | Description | MIBiG Cluster | MiBiG Product | % ID | % Coverage | BLAST Score | E-value |
| --- | --- | --- | --- | --- | --- | --- | --- |
| AFV52183.1 | aldolase | BGC0000081 | Kedarcidin\_biosynthetic\_gene\_cluster | 46 | 81.8181818182 | 225 | 8.3e-59 |
| EWG54271.1 | hypothetical\_protein | BGC0001190 | Fusaric\_acid\_biosynthetic\_gene\_cluster | 42 | 83.8383838384 | 216 | 5e-56 |
| AAF67511.1 | NovR | BGC0000834 | Novobiocin\_biosynthetic\_gene\_cluster | 42 | 87.8787878788 | 210 | 2.8e-54 |
| AAN65240.1 | putative\_aldolase | BGC0000832 | Clorobiocin\_biosynthetic\_gene\_cluster | 42 | 89.2255892256 | 208 | 8e-54 |
| ADY16691.1 | TqaM | BGC0001142 | Tryptoquialanine\_biosynthetic\_gene\_cluster | 37 | 92.9292929293 | 208 | 8e-54 |
